# Identifying “hot papers” and papers with “delayed recognition” in large-scale datasets by using dynamically normalized citation impact scores

**DOI:** 10.1007/s11192-018-2772-0

**Published:** 2018-05-19

**Authors:** Lutz Bornmann, Adam Y. Ye, Fred Y. Ye

**Affiliations:** 1 Division for Science and Innovation Studies, Administrative Headquarters of the Max Planck Society, Hofgartenstr. 8, 80539 Munich, Germany; 2 Center for Bioinformatics, School of Life Sciences, Peking University, Beijing, 100871 China; 3 Jiangsu Key Laboratory of Data Engineering and Knowledge Service, Nanjing University, Nanjing, 210023 China

**Keywords:** Hot paper, Paper with delayed recognition, Field-normalized impact scores

## Abstract

“Hot papers” (HPs) are papers which received a boost of citations shortly after publication. Papers with “delayed recognition” (DRs) received scarcely impact over a long time period, before a considerable citation boost started. DRs have attracted a lot of attention in scientometrics and beyond. Based on a comprehensive dataset with more than 5,000,000 papers published between 1980 and 1990, we identified HPs and DRs. In contrast to many other studies on DRs, which are based on raw citation counts, we calculated dynamically field-normalized impact scores for the search of HPs and DRs. This study is intended to investigate the differences between HPs (*n* = 323) and DRs (*n* = 315). The investigation of the journals which have published HPs and DRs revealed that some journals (e.g. *Physical Review Letters* and PNAS) were able to publish significantly more HPs than other journals. This pattern did not appear in DRs. Many HPs and DRs have been published by authors from the USA; however, in contrast to other countries, authors from the USA have published statistically significantly more HPs than DRs. Whereas “Biochemistry & Molecular Biology,” “Immunology,” and “Cell Biology” have published significantly more HPs than DRs, the opposite result arrived for “Surgery” and “Orthopedics.” The results of the analysis of certain properties of HPs and DRs (e.g. number of pages) suggest that the emergence of DRs is an unpredictable process.

## Introduction

In most evaluations of researchers, research groups, and academic institutions, bibliometric indicators—especially citation impact scores—are used in an informed peer review process (Bornmann et al. 2014). A frequent problem of the application of citation impact scores in these processes is that the evaluations focus—as a rule—on the recent performance of the evaluated units (e.g. the last 3 years). However, the “true” impact of a publication can be determined only after a longer time period in several disciplines: “A 3-year time window is sufficient for the biomedical research fields and multidisciplinary sciences, while a 7-year time window is required for the humanities and mathematics” (Wang 2013, p. 866). Thus, the strength of bibliometrics entails identifying outstanding publications (or the corresponding outstanding researchers, research groups, and institutions, respectively) in the long term.

In recent years, several bibliometric studies have dealt with the investigation of a sub-group of publications showing a specific long term citation impact: papers with delayed recognition (DRs). Publications are denoted as DRs if they received only a few or no citations over many years (e.g., 10 years after their appearance) and then experienced a significant boost in citations. For example, Van Calster (2012) shows that Charles Sanders Peirce (1884) note in *Science* on “The Numerical Measure of the Success of Predictors” is a typical case of a DR. The note received “less than 1 citation per year in the decades prior to 2000, 3.5 citations per year in the 2000s, and 10.4 in the 2010s” (p. 2342). Marx (2014) demonstrates that the initial reception of the paper “Detailed Balance Limit of Efficiency of P–N Junction Solar Cells” by Shockley and Queisser (1961) was hesitant; after several years, the paper has become a highly cited paper in its field. Gorry and Ragouet (2016) present a landmark paper in interventional radiology, which can be characterized as a DR.

In “Literature review” section, we explain the different methods which have been introduced in scientometrics to identify these and other DRs in bibliometric databases. Based on these methods, Ye and Bornmann (2018) propose the citation angle, which can be used to distinguish between “hot papers” (HPs) and DRs. In contrast to DRs, HPs received a boost of citations shortly after publication (and not after several years as DRs). In this study, we searched for HPs and DRs among all papers published between 1980 and 1990. Since citation counts should be normalized with regard to publication year and subject category (of the cited publication), we generated dynamically normalized citation impact scores (DNIC), which are annually field-normalized impact scores based on OECD minor codes^1^ for field delineation. We used these scores for the search of HPs and DRs. The objective of this study is to analyze systematic differences between papers which became HPs or DRs later on. Factors which have been identified in recent years as correlates of citations (Bornmann & Leydesdorff 2017; Tahamtan, Safipour Afshar, & Ahamdzadeh, 2016) are used to determine different characteristics of both paper groups. As factors, this study focuses on the publication year, the number of authors, countries, references and pages of a publication as well as its inter-disciplinarity (measured by the number of subject categories).

## Literature review

Early engagement in DRs started with the pioneering works of Garfield (1970, 1980, 1989a, b, 1990). Furthermore, Stent (1972) discusses “prematurity” in scientific discovery. Large-scale empirical analysis of delayed recognition started with Glänzel et al. (2003) who analyzed early papers from 1980. They assigned papers the attribute “delayed recognition” if they received 1 or 2 citations in the first years and at least 100 citations later on. They identified less than 100 papers with this citation profile. With a slightly changing definition of “delayed recognition,” which considers the Journal Impact Factor (JIF), Glänzel and Garfield (2004) found that 1.3 per 10,000 papers were neglected initially, but are highly cited later on. The JIF is the mean citations within 1 year, which have been published in the two previous years.

van Raan (2004b) introduced the term “sleeping beauty” for DRs. He suggests the following criteria for identifying DRs: (1) Depth of sleep (*c*_s_): the paper received at most 1 citation per year on average (very deep sleep) or between 1 and 2 citations per year on average (deep sleep) after its appearance. (2) Length of sleep (*s*): the length of the sleeping period. (3) The awakening intensity (*c*_*w*_): the annual citations during the 4 years period following the sleep. van Raan (2004b) developed the so called Grand Sleeping Beauty Equation for estimating the number of DRs: $N = f\left\{ {s,c_{\text{s}} ,c_{w} } \right\}\,{\sim}\,s^{ - 2.7} c_{\text{s}}^{2.5} c_{w}^{ - 6.6}$, where *N* is the number of DRs.

Costas et al. (2010) defined various types of citation profiles: “Yr 50%” is that year by when a paper has received at least 50% of its citations in the corresponding subject categories and publication years. “P25” and “P75” denote the prior 25 and 75% citations as one-fourth and three-fourth quartile criterions. According to Costas et al. (2010), “flashes in the pan” can be defined “as those documents that have received 50% of their citations when the 75% of other documents still have not received 50% of their citations. Normal documents are all documents that receive the 50% of their citations around the year of P50 (between P25 and P75). Finally, delayed documents are those papers that have received 50% of their citations after P75 years in their fields” (p. 331).

Li and Ye (2012) introduced the term “all-elements-sleeping-beauties” (ASBs). The term is intended for publications for which “spindles, sleeping beauties, and princes” co-exist. In a follow-up paper, Li et al. (2014) introduced additionally the “heartbeat spectra” of DRs. Whereas the “heartbeat” defines the annual citations of DRs in the sleeping period, the “heartbeat spectrum” describes the vector of the DR’s heartbeat. If *c*_*i*_ denotes the citation counts which the DR received in the *i*th year of the sleeping period, the DR’s heartbeat in the *i*th year is *c*_*i*_. Then, vector *H* = (*c*_*1*_,…, *c*_*i*_,…, *c*_*n*_) is the heartbeat spectrum, in which *n* indicates the duration of the sleeping period. Two further studies (Huang et al. 2015; Li and Shi 2016) in this series of studies which started with Li and Ye (2012) deal with the awakening of DRs.

Ke et al. (2015) introduced the beauty coefficient *B* for the identification of DRs. The coefficient *B* is defined as follows (for the purpose of simplifying the formula, we use *c*_*m*_ instead of *c*_*tm*_):

1 $$B = \sum\limits_{{t = t_{0} }}^{{t_{m} }} {\frac{{\frac{{c_{\text{m}} - c_{0} }}{{t_{m} }}t + c_{0} - c_{t} }}{{\hbox{max} \{ 1,c_{t} \} }}}$$

where *c*_*t*_ is the citation counts received in the *t*th year after publication and *t* the age of a paper. A paper reached the maximum number *c*_*m*_ of annual citations at time *t*_*m*_. The equation of the straight line (*l*) which connects two points (0, *c*_*0*_) and *(t*_*m*_*, c*_*m*_) in the annual citation curve is defined as

2 $$l:c = \frac{{c_{m} - c_{0} }}{{t_{m} }}t + c_{0} .$$

Cressey (2015) assumes that the coefficient *B* is an elegant and effective method for DRs retrievals in big datasets. Ye and Bornmann (2018) reveal its dynamic characteristics and extend *B* by a HP component. Furthermore, they introduced the citation angle for unifying the approaches of identifying instant and delayed recognition. The distinction between DRs and HPs follows Baumgartner and Leydesdorff (2014) who introduced two groups of papers: (1) “Citation classics” or “sticky knowledge claims” have a lasting impact on a specific field. DRs are a sub-group among citation classics, whose lasting impact is not combined with early citation impact. (2) The other paper group (“transient knowledge claims”) has an early boost of citation impact followed by a fast impact decrease shortly after publication. According to Baumgartner and Leydesdorff (2014) the papers in this group are contributions at the research front. Comins and Leydesdorff (2016) investigated the existence of both paper types empirically.

van Raan (2015) demonstrated that many DRs are application-oriented and thus are potential “sleeping innovations”. In a follow-up study, van Raan (2016) analyzed characteristics of DRs which are cited in patents. The results show that patent citations occur before or after the delayed recognition started. The citation rate during the period of sleep is not related to the later scientific or technological impact of the DRs. The comparison of DRs with “normal” papers reveals that DRs are more frequently cited in patents than “normal” papers.

## Methods

### Definitions of “hot papers” (HP) and papers with “delayed recognition” (DRs)

Following the definitions of HPs and DRs hitherto, the typical DR is defined as a publication with a late citation peak, and prior annual citations which are much lower than the peak citations, while a typical HP is defined as a publication with an early citation peak and later annual citations which are much lower than the early peak. In contrast to the other studies, which used raw citation counts to identify DRs (see “Literature review” section), this study is based on (dynamically) field- and time-normalized citation impact scores—the standard impact measure in bibliometrics (Vinkler, 2010). The dynamically normalized impact of citations (DNIC) is defined as

3 $${\text{DNIC}}_{ij} = \frac{{C_{ij} }}{{E_{kj} }},\quad k = f(i)$$

4 $$E_{kj} = \frac{1}{{N_{kj} }}\sum\limits_{{i\left| {k = f(i)} \right.}} {C_{ij} }$$

where *i *= 1,2,… are publications, *j *= 1,2,… are citing years, and *k *= 1,2,… are different fields (here defined by OECD minor codes). *C*_*ij*_ denotes received citations by publication *i* in year *j,* and *E*_*kj*_ denotes mean (received) citations of all publications in field *k* and year *j* (i.e. *E*_*kj*_ is the expected value). *N*_*kj*_ is the number of cited publications in field *k* and year *j* (note: *N*_*kj*_ is a variable which is based on non-zero citations), and *k *= f(*i*) means a certain field of a given publication. The indicator follows the standard approach in bibliometrics with both field- and time-normalized citations (Waltman 2016). The only difference to the standard approach is that the calculation is based on annual citations (dynamically), but not on the citations between publication year and a fixed time point later on. If *C*_*ij*_ = 0, then DNIC_*ij*_ = 0.

**Fig. 1:**
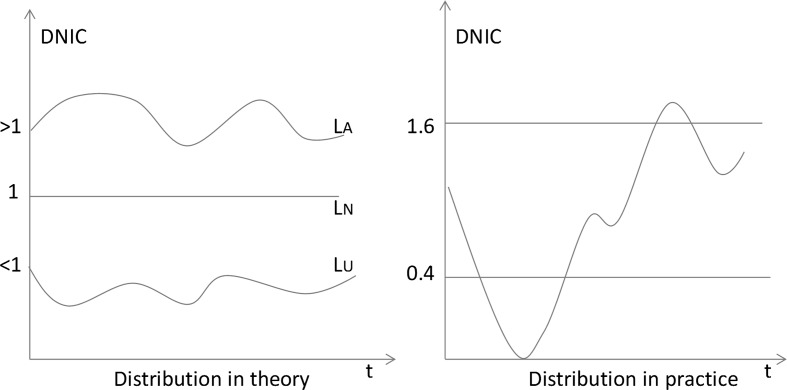
Distributions in theory and in practice of dynamically normalized citation impact scores (DNIC)

All points of DNIC_*ij*_ = 1 in field *k* yield the field- and time- normalized line *L*_*N*_ (see the distribution in theory of DNIC in Fig. 1). If DNIC_*ij*_ > 1, the citation impact of the publications is higher than the average in the corresponding fields and publication years, as shown with line *L*_*A*_. If DNIC_*ij*_ < 1, the impact is lower than the average, as shown with the line *L*_*U*_. In practical terms, however, citation counts *C*_*ij*_ and expected values *E*_*kj*_ are variable terms. The DNIC distribution of many papers changes from year to year (see the distribution in practice in Fig. 1). Therefore, by using DNIC for impact normalization of papers in this study we need rules for identifying HPs and DRs. We oriented these rules towards the rules of thumbs defined by van Raan (2004a, 2008) for interpreting field-normalized citation scores. DNIC_*ij*_ is a dynamic series of annually normalized impact scores. We suggest identifying HPs and DRs with the criteria given in Table 1.

**Table 1:** Criteria used in this study for identifying HPs and DRs

Citation profile	DNIC_peak*_t*<*th*_	DNIC_*a_*peak*_t*_	Peak*_t*	DNIC_*b_*peak*_t*_	DNIC_peak*_t*>*th*_
HP	> 2	< .4	<* t*_*h*_		
DR			>* t*_*h*_	< .4	>1.6

In Table 1, DNIC_peak*_t*<*th*_ denotes that the peak is located in the early-half time span of the citation impact distribution (covering ± 2 years); DNIC_peak*_t*>*th*_ denotes that the peak is located in the late-half time span (covering ± 2 years). DNIC_*a_*peak*_t*_ refers to all DNIC_*ij*_ after the peak (+ 2 years), and DNIC_*b_*peak*_t*_ refers to all DNIC_*ij*_ before the peak (− 2 years). In this study, *t*_*h*_ = 13. We have data covering 36 citing years (1980–2015) and needed to compare the years 1980–1990 dynamically. Thus, we selected 16 years as the time span of citations for each publication, such as 1980–1995 for the papers from 1980 and 1981–1996 for the papers from 1981.

### Used datasets

Table 2 shows the number of papers from 1980 to 1990 which have been considered in this study. The bibliometric data are from an in-house database developed and maintained by the Max Planck Digital Library (MPDL, Munich). The in-house database is based on the Web of Science (WoS, Clarivate Analytics, formerly the IP & Science business of Thomson Reuters). From the in-house database, we selected only papers with the document type “article” to have comparable citable units. The DNIC scores for each paper refer to the period from its publication year until the end of 2015.

**Table 2:** Numbers of identified HPs and DRs from the total number of articles

Publication year	HP	DR	Total number of articles
1980	177	21	402,417
1981	181	17	423,754
1982	203	36	438,201
1983	203	20	464,131
1984	211	33	479,977
1985	213	41	495,496
1986	191	50	508,608
1987	178	40	524,467
1988	252	42	539,656
1989	254	26	558,316
1990	204	43	536,566
Total	2267	369	5,371,589

**Fig. 2:**
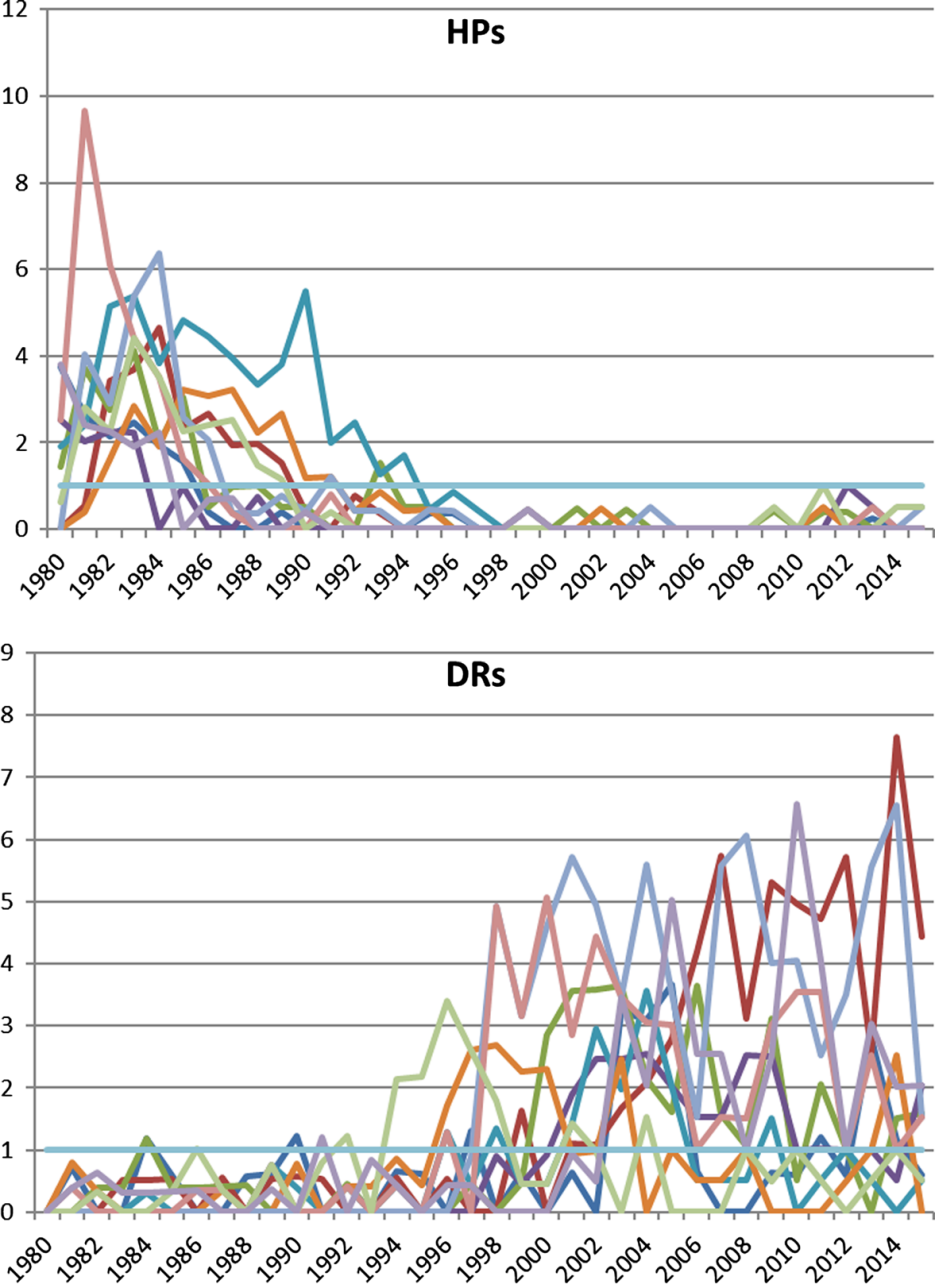
DNCI of HPs and DRs in our dataset published in 1980

Using the methods explained in “Definitions of ‘hot papers’ (HP) and papers with ‘delayed recognition’ (DRs)” section, we found the numbers of HPs and DRs in the dataset as reported in Table 2. Since HPs and DRs have been identified by using normalized impact scores within single fields and many papers belong to more than one field, there are duplicates among HPs and DRs. Thus, 191 duplicates were deleted of the 2636 DRs and HPs (147 papers were twice and 44 papers three times in the dataset). Figure 2 demonstrates clear differences in citation profiles of HPs and DRs following the definitions of both groups in “Definitions of ‘hot papers’ (HP) and papers with ‘delayed recognition’ (DRs)” section.

Both, HPs and DRs are groups of papers with extreme citation profiles (see Fig. 2). In order to reveal how these extreme groups differ from “normal” papers in certain properties, we drew a random sample from the in-house database with *n *= 323 papers (date December 8, 2016). The random sample has been selected in those WoS subject categories in which most of the DRs and HPs were published (i.e., the ten subject categories in which most of the DRs and HPs were published). The population of the random sample (*N *= 1,198,843) contains papers from 1980 to 1990 and is restricted to the document type “article”. The size of the random sample with *n* = 323 papers has been determined by a power analysis. Its results showed that we need 323 papers in each group to detect a very small effect, *f *= .1 (Cohen, 1988), as statistically significant at the *α* = .05 level with a power of .8 (Acock 2016).

Considering the third group of randomly selected papers (RANs), the dataset (*n *= 2768) of this study consists of 2130 HPs (77%), 315 DRs (11%), and 323 RANs (12%). In order to have three groups of papers with a more or less balanced set of case numbers, we drew a random sample of 323 papers from the 2130 HPs—following the results of the power analysis. Thus, the final dataset (*n *= 961) consists of 323 HPs (33.6%), 315 DRs (32.8%), and 323 RANs (33.6%).

### Statistical methods

This study tests whether the mean values (e.g., the mean number of authors or pages) from *k* groups (HP, DR, and RAN) are the same or not. With the analysis of variance (ANOVA) any overall difference between the *k* groups can be tested on statistical significance. The ANOVA separates the variance components into two parts: those due to mean differences and those due to random influences (Riffenburgh, 2012). There are three general assumptions for calculating the ANOVA: (1) The data are independent of each other. (2) The distribution of the data is normal. (3) The standard deviation of the data is the same for all groups (HP, DR, and RAN). Although these assumptions are violated here, the ANOVA is still applied: according to Riffenburgh (2012), the ANOVA “is fairly robust against these assumptions” (p. 265), especially in those studies in which the sample size is high. In order to counter-check the results of the ANOVA, the Kruskal–Wallis rank test (KW test) has been additionally applied as the non-parametric alternative (Acock 2016).

The effect size eta squared (*η*^*2*^) is additionally calculated to the ANOVA which is a measure of the practical significance of the results (Acock 2016). Eta squared is the sum of squares for a factor (here: three groups of papers with different citation profiles) divided by the total sum of squares. The effect size shows how much of the variation in the sample of papers (e.g. with respect to the number of authors) is explained by the factor. According to Cohen (1988), a value of *η*^2^ = .01 means a small effect, *η*^2^ = .06 a medium effect, and *η*^2^ = .14 a large effect. The consideration of the practical significance is especially important in studies in which the case numbers are high (Kline 2004). There is a risk in these studies that the results of statistical tests are significant although the effects (e.g., mean differences between *k* groups) are small.

Beyond the ANOVA, the *t* test is applied in this study to undertake pairwise comparisons of group means. Thus, it is not only tested whether the mean differences between all *k* groups (where *k* > 2) are statistically significant, but also the mean differences between the specific pairs of groups. The *t* test is seen as a very robust statistic; for the *t* test, however, the same assumptions hold as for the ANOVA (see above). Since the assumptions are not fulfilled in each calculation here, the non-parametric alternative referred to as the Mann–Whitney two-sample rank-sum test is additionally used (Acock 2016). For multiple pairwise comparisons, the chance of the likelihood of incorrectly rejecting the null hypothesis increases. Thus, the Bonferroni correction is used which compensates for that by testing each pairwise comparison at a significance level of .05/3 = .017 (.05 is the alpha level and 3 is the number of pairwise comparisons). As a measure of effect size in addition to the *t* test, Cohen’s *d* is applied. For Cohen (1988), *d* = .2 is a small effect, *d *= .5 a moderate effect, and *d* = .8 a large effect.

The Chi Square test of independence is used in this study to determine if there is a significant association between two nominal (categorical) variables. The frequency of a specific nominal variable is compared with different values of a second nominal variable. The required data can be shown in an R*C contingency table, where *R* is the row and *C* is the column.

### Factors with an influence on citation counts (FICs)

In recent years, many different factors have been identified which may influence the number of citations a publication receives. Although these factors turn out to be correlated with citations and causality cannot be assumed (Bornmann and Leydesdorff 2017), they are generally considered to be influencing factors. On a given time axis, the citations follow the appearance of a publication with specific characteristics (e.g., a specific number of authors or pages). However, one should have in mind for this perspective on the factors that moderating factors might exist. For example, the JIF might count as FIC; however, high citation counts for papers published in high-impact journals could be the result of the quality of the papers which influence both, the JIF of the publishing journals as well as the number of citations.

In the last years, several studies have been published investigating the relationship between number of pages and citations of papers in different disciplines. Stanek (2008) found that for papers published in astronomy journals their length is associated with the number of citations they received. The same result is reported by Leimu and Koricheva (2005) for ecological papers, by Hegarty and Walton (2012) for psychology papers, and by Gillmor (1975) for the *Journal of Atmospheric and Terrestrial Physics*. Similar results have been published also by several other authors for various other disciplines (Beaver 2004; Fok and Franses 2007; Lawani 1986; Tregenza 2002; Vanclay 2013; Wesel et al. 2013). The most important reason for the correlation between both variables might be that longer papers contain more citable content.

Similar to the number of pages, the number of cited references of a paper seems also be related to the number of citations this paper receives. Webster et al. (2009) found that reference counts explain 19% of the variance in citation counts. In psychology, reference list length predicts citation impact better than the JIF of the publishing journal (Hegarty and Walton 2012). The JIF is generally seen as the FIC with the most predictive power (Onodera and Yoshikane 2014). For several disciplines, Wesel et al. (2013) report positive correlations between the number of cited references and citation counts. Similar results have been published by Fok and Franses (2007) and Onodera and Yoshikane (2014). Webster et al. (2009) provide the following reasons for the correlation between both variables: “First, review articles (e.g., theoretical reviews, meta-analyses) tend to have more citations than and are cited more frequently than typical empirical articles. Second, scientists are humans, and humans crave recognition for their work and often participate in reciprocal altruism … The more people you cite in your paper, the more people are likely to cite your paper (the paper they were cited in) in the future. Third, the Matthew effect—the idea that ‘the rich get richer,’ that publications that are initially highly cited tend to have the advantage of being cited even more in the future—may also occur” (p. 349).

Besides the JIF, the number of authors is seen as another important FIC. Leimu and Koricheva (2005) found for ecological papers that “papers with four or more authors received more citations than did papers with fewer authors” (p. 30). Similar results have been reported by Robson and Mousquès (2016) for environmental modeling papers and by Wesel et al. (2013) for several other disciplines. According to the case study by Mirnezami et al. (2016) including researchers in Quebec (Canada), researchers who publish within larger teams of authors receive also more citation impact. There might be several reasons for the association between number of authors and number of citations: “We can think of a reference by n authors as having n times more proponents than a solo-authored one. This would include self-citations in other papers (as already observed in the study), citations in other kinds of scientific literature, and an increased number of research groups being familiar with the article. Moreover, scientific communication is not limited to journals. The longer the author list is, the greater the probability of the paper being presented to several conferences is, especially if the team is multidisciplinary” (Valderas 2007).

Iribarren-Maestro et al. (2007) investigated papers published by Carlos III University of Madrid (Spain) researchers. They found that the number of countries is correlated with the number of citations the papers received. Furthermore, there are empirical evidences that interdisciplinary research receives more citation impact than disciplinary research (Haustein et al. 2014).

## Results

Before we come in “Factors with an influence on citation counts (FICs)” section to the FICs and their relationship to HPs and DRs, we show in “Publishing journals and overall citation impact” section possible differences between both groups concerning their publishing journal and overall citation impact.

### Publishing journals and overall citation impact

Table 3 shows the journals in which at least ten HPs and DRs appeared. Whereas only one journal has published more than 10 DRs (*Clinical Orthopaedics and Related Research*), there are five journals in the list of HPs. A closer inspection of the list of journals publishing at least 25% of the HP and DR papers, respectively, showed that these are 16 for DRs and only 8 for HPs. Since we found 19 journals which represent 25% of the RAN papers, the number of journals for the DRs is similar to what can be expected by chance.

**Table 3:** Journals in which at least ten HPs and DRs appeared

Type	Journal	Number of papers	Percent
HP	Journal of Biological Chemistry	14	4.33
	Physical Review Letters	13	4.02
	PNAS	13	4.02
	Journal of Immunology	11	3.41
	Genomics	10	3.10
DR	Clinical Orthopaedics and Related Research	29	9.21

How do the three types of citation profiles differ in terms of their overall citation impact? To answer this question, we used the field-normalized citation impact scores named as Mean Normalized Citation Score (MNCS) (Waltman et al. 2011a, b). Here, the citation impact of the focal paper is divided by the mean citation impact in the corresponding field. A variant of the MNCS does not normalize the citation impact on the entire field, but on the journal, in which the focal papers was published. The relation of a publication’s MNCS and DNIC is characterized by ${\text{MNCS}}_{\text{j}} = \sum\nolimits_{i} {{\text{DNIC}}_{ij} }$. We expected that HPs and DRs are characterized by high scores in terms of normalized impact, since both types produced impact either in the short or in the long term.

The results in Table 4 confirm our expectations: Whereas the randomly selected papers show mean MNCS scores which correspond to an “average” impact in a field or journal, respectively, both citation profile types, HPs and DRs, have scores which are significantly above the average. Especially DRs are concerned by very high impact scores. Thus, the papers of both paper types should be identifiable as high-impact using the standard advanced bibliometric indicators.

**Table 4:** Mean differences in MNCS (based on the entire field or publishing journal) between DRs, HPs, and RANs

Citation profile	Mean	Standard deviation	Median	Minimum	Maximum	Number of papers
*MNCS (field)*						
HP	2.6^a,b^	1.2	2.3	.8	6.9	323
DR	5.5^a,c^	5.6	4	.9	56	315
RAN	1.0^b,c^	2.6	.2	0	28.4	323
Total	3.0	4.1	2.2	0	56	961
*MNCS (journal)*						
HP	1.9^a,b^	1.7	1.4	.2	11.3	323
DR	5.5^a,c^	7.6	3.4	.3	79	315
RAN	1.1^b,c^	2.3	.4	0	22	323
Total	2.8	5	1.5	0	79	961

MNCS (field):

*F*(2, 960) = 125.61, *p *= .000, *η*^2^ = .21 [.16, .25], *χ*^2^(2) = 518.91, *p* = .000

Pairwise comparisons:

^a^*t*(1, 636) = − 9.12, *p* = .000, *d* = − .72 [− .88, − .56], *z* = − 12.09, *p* = .000

^b^*t*(1, 644) = 9.92, *p* = .000, *d* = .78 [.62, .94], *z* = 17.46, *p* = .000

^c^*t*(1, 636) = 12.96, *p* = .000, *d* = 1.03 [.86, 1.19], *z* = 19.07, *p* = .000

MNCS (journal):

*F*(2, 960) = 81.59, *p *= .000, *η*^2^ = .15 [.11, .19], *χ*^2^(2) = 421.25, *p* = .000

Pairwise comparisons:

^a^*t*(1, 636) = − 8.38, *p* = .000, *d* = − .66 [− .82, − .50], *z* = − 13.48, *p* = .000

^b^*t*(1, 644) = 4.95, *p* = .000, *d* = .39 [.23, .55], *z* = 12.51, *p* = .000

^c^*t*(1, 636) = 10.00, *p* = .000, *d* = .79 [.63, .95], *z* = 17.80, *p* = .000

### Factors with an influence on citation counts (FICs)

With publication year, number of pages, number of references, number of authors, number of countries, and number of subject categories, factors are considered here, which have been (frequently) investigated in former studies. Overviews on studies investigating FICs can be found in Peters and van Raan (1994), Onodera and Yoshikane (2014), Didegah and Thelwall (2013), and Bornmann and Daniel (2008). The results of the studies indicate that publication year, number of pages, number of references, number of authors, number of countries, and number of subject categories are regarded as possible FICs.

The first FIC which we look at in this study is the publication year of the cited paper (Ruano-Ravina and Alvarez-Dardet 2012). Besides the journal or field, respectively, in which a publication appeared the publication year is generally considered in the normalization of citations (Waltman 2016). Since DRs emerge in the long term, we expected an earlier mean publication year for DRs than for HPs. However, the results in Table 5 show that the empirical evidence looks differently: With *M *= 1985.2, HPs have been published similarly on average as DRs (*M *= 1985.6). Furthermore, the differences between the three groups (HP, DR, and RAN) are statistically not significant and the effect sizes are very low. The negligible differences in Table 5 are certainly the result of the use of normalized impact scores for the identification of HPs and DRs. Thus, the results in the table confirm the effectiveness of the normalization procedure used in this study.

**Table 5:** Mean differences in publication years between HPs, DRs, and RANs

Citation profile	Mean	Standard deviation	Median	Minimum	Maximum	Number of papers
HP	1985.2^a,b^	3.2	1985	1980	1990	323
DR	1985.6^a,c^	3	1986	1980	1990	315
RAN	1985.4^b,c^	3.1	1986	1980	1990	323
Total	1985.4	3.1	1986	1980	1990	961

*F*(2, 960) = 1.20, *p *= .301, *η*^2^ = .00 [.00, .01], *χ*^2^(2) = 2.00, *p* = .37

Pairwise comparisons:

^a^*t*(1, 636) = − 1.55, *p* = .12, *d* = − .12 [− .28, .03], *z* = − 1.46, *p* = .14

^b^*t*(1, 644) = − .79, *p* = .43, *d* = − .06 [− .22, .09], *z* = − .70, *p* = .48

^c^*t*(1, 636) = .77, *p* = .45, *d* = .06 [− .09, .22], *z* = .64, *p* = .52

Table 6 shows the differences in the number of pages between HPs, DRs, and RANs. DRs (*M *= 9.6, MDN = 8) have more pages than HPs (*M *= 8.2, MDN = 7) and RANs (*M *= 7.3, MDN = 6). However, the reported effect sizes in the table are small in general.

**Table 6:** Mean differences in number of pages between DRs, HPs, and RANs

Citation profile	Mean	Standard deviation	Median	Minimum	Maximum	Number of papers
HP	8.2^a,b^	6.3	7	2	55	320
DR	9.6^a,c^	9.2	8	2	133	314
RAN	7.3^b,c^	4.9	6	2	28	316
Total	8.3	7.1	7	2	133	950

*F*(2, 947) = 8.30, *p *= .000, *η*^2^ = .02 [.003, .04], *χ*^2^(2) = 30.51, *p* = .000

Pairwise comparisons:

^a^*t*(1, 632) = − 2.25, *p* = .03 (n.s.), *d* = − .18 [− .33, − .02], *z* = − 3.42, *p* = .001

^b^*t*(1, 634) = 1.91, *p* = .06, *d* = .15 [− .01, .31], *z* = 2.28, *p* = .02 (n.s.)

^c^*t*(1, 628) = 3.85, *p* = .000, *d* = .31 [.15, .46], *z* = 5.42, *p* = .000

Table 7 shows mean differences in (linked) cited references between HPs, DRs, and RANs. The table reports the results of two analyses. The first section in the table refers to all cited references in the papers. The results in the second section are based on a sub-group of all cited references: the linked cited references could be matched with publication records in the WoS in-house database (i.e., with publications from journals covered in WoS). The results in Table 7 show that HPs have included statistically significantly more (linked) cited references than DRs and RANs. DRs are based on a similar number of linked cited references as RANs.

**Table 7:** Mean differences in (linked) cited references between HPs, DRs, and RANs

Citation profile	Mean	Standard deviation	Median	Minimum	Maximum	Number of papers
*Cited references*						
HP	29.8^a,b^	20.1	26	0	162	323
DR	21.9^a,c^	23.8	17	0	229	315
RAN	17.3^b,c^	14.7	15	0	91	323
Total	23.0	20.5	20	0	229	961
*Linked cited references*						
HP	15.4^a,b^	14.5	13	0	121	323
DR	5.1^a,c^	8.5	3	0	91	315
RAN	6.8^b,c^	8.8	3	0	43	323
Total	9.1	11.8	5	0	121	961

Cited references:

*F*(2, 958) = 32.73, *p *= .000, *η*^2^ = .06 [.04, .10], *χ*^2^(2) = 106.30, *p* = .000

Pairwise comparisons:

^a^*t*(1, 636) = 4.52, *p* = .000, *d* = .36 [.20, .51], *z* = 7.60, *p* = .000

^b^*t*(1, 644) = 9.03, *p* = .000, *d* = .71 [.55, .87], *z* = 9.64, *p* = .000

^c^*t*(1, 636) = 2.96, *p* = .003, *d* = .24 [.08, .39], *z* = 2.94, *p* = .003

Linked cited references:

*F*(2, 958) = 80.90, *p *= .000, *η*^2^ = .15 [.11, .18], *χ*^2^(2) = 171.06, *p* = .000

Pairwise comparisons:

^a^*t*(1, 636) = 10.91, *p* = .000, *d* = .86 [.70, 1.03], *z* = 12.50, *p* = .000

^b^*t*(1, 644) = 9.05, *p* = .000, *d* = .71 [.55, .87], *z* = 9.98, *p* = .000

^c^*t*(1, 636) = − 2.56, *p* = .01, *d* = − .20 [− .36, − .05], *z* = − 1.53, *p* = .13

The mean differences in number of authors between HPs, DRs, and RANs are shown in Table 8. The mean number of authors for HPs (*M *= 4.8) is high compared to DRs (*M *= 2.6) and RANs (*M *= 2.7). The effect sizes of the results are medium.

**Table 8:** Mean differences in number of authors between HPs, DRs, and RANs (one paper with zero authors has been excluded)

Citation profile	Mean	Standard deviation	Median	Minimum	Maximum	Number of papers
HP	4.8^a,b^	5.5	4	1	63	323
DR	2.6^a,c^	1.6	2	1	12	315
RAN	2.7^b,c^	1.8	2	1	14	322
Total	3.4	3.6	3	1	63	960

*F*(2, 957) = 40.15, *p *= .000, *η*^2^ = .08 [.05, .11], *χ*^2^(2) = 104.85, *p* = .000

Pairwise comparisons:

^a^*t*(1, 636) = 6.80, *p* = .000, *d* = .54 [.38, .70], *z* = 9.37, *p* = .000

^b^*t*(1, 643) = 6.38, *p* = .000, *d* = .50 [.35, .66], *z* = 8.61, *p* = .000

^c^*t*(1, 635) = − 1.02, *p* = .31, *d* = − .08 [− .24, .07], *z* = − .64, *p* = .52

Since the affiliation information on the papers contains the country of the authors where they are working, we can investigate whether certain countries are especially associated with the publication of HPs and DRs and whether there are mean differences in the number of countries per paper between HPs, DRs, and RANs. Table 9 shows the ten countries with the most DRs and HPs. With *n* = 333 papers, significantly more HPs and DRs have been published by authors from the USA than from other countries. This result is not surprising and in agreement with most other country-specific statistics including all publications (National Science Board 2016). It follows Great Britain (*n* = 76), Japan (*n *= 42), and Germany (*n* = 39). The USA is the only country in Table 9 with a statistically significant difference in the number of HPs and DRs: With *n* = 194, significantly more HPs have been published by authors from the USA than DRs (with *n *= 139).

**Table 9:** Ten countries with the most HPs and DRs

Country		HP	DR	Total	*χ*^2^(1)/*p*
USA	Absolute	194	139	333	16.23
	In percent	58.26	41.74	100	**.001**
Great Britain	Absolute	46	30	76	3.38
	In percent	60.53	39.47	100	.66
Japan	Absolute	23	19	42	.31
	In percent	54.76	45.24	100	1.00
Germany	Absolute	23	16	39	1.16
	In percent	58.97	41.03	100	1.00
France	Absolute	20	9	29	4.09
	In percent	68.97	31.03	100	.43
Canada	Absolute	12	15	27	.43
	In percent	44.44	55.56	100	1.00
Italy	Absolute	4	14	18	5.98
	In percent	22.22	77.78	100	.15
The Netherlands	Absolute	12	5	17	2.78
	In percent	70.59	29.41	100	.952
Australia	Absolute	8	9	17	.09
	In percent	47.06	52.94	100	1.00
Switzerland	Absolute	10	6	16	.93
	In percent	62.5	37.5	100	1.00

The table shows absolute and relative numbers as well as Pearson *χ*^2^ values with Bonferroni-adjusted *p* values (the statistically significant result is printed in bold)

Table 10 shows mean differences in number of countries between HPs, DRs, and RANs. We tested the mean difference since there are evidences that the number of countries is related to the number of citations (see above). However, our results in Table 10 reveal that the number of countries does not discriminate between the three groups. The practical significances are small.

**Table 10:** Mean differences in number of countries between HPs, DRs, and RANs (papers with zero countries have been excluded)

Citation profile	Mean	Standard deviation	Median	Minimum	Maximum	Number of papers
HP	1.2^a,b^	.5	1	1	4	323
DR	1.1^a,c^	.3	1	1	3	311
RAN	1.1^b,c^	.3	1	1	3	312
Total	1.1	.4	1	1	4	946

*F*(2, 943) = 4.24, *p *= .02, *η*^2^ = .01 [.000, .02], *χ*^2^(2) = 1.63, *p* = .44

Pairwise comparisons:

^a^*t*(1, 632) = 1.85, *p* = .06, *d* = .15 [− .01, .30], *z* = 1.49, *p* = .14

^b^*t*(1, 633) = 2.69, *p* = .01, *d* = .21 [.06, .37], *z* = 2.35, *p* = .02 (n.s.)

^c^*t*(1, 621) = .18, *p* = .35, *d* = .08 [− .08, .23], *z* = .87, *p* = .38

As a last FIC in this study, we investigated the number of subject categories. The number of subject categories for a paper can be used as an indicator of inter-disciplinarity. We used the WoS subject categories which have been assigned by Clarivate Analytics to the papers on the base of the publishing journals. Table 11 shows the mean differences in number of subject categories between HPs, DRs, and RANs. As the results reveal, the differences are of no practical relevance.

**Table 11:** Mean differences in number of subject categories (as a measure of inter-disciplinarity) between HPs, DRs, and RANs

Citation profile	Mean	Standard deviation	Median	Minimum	Maximum	Number of papers
HP	1.5^a,b^	.7	1	1	4	323
DR	1.7^a,c^	.8	1	1	5	315
RAN	1.5^b,c^	.8	1	1	5	323
Total	1.5	.8	1	1	5	961

*F*(2, 958) = 4.88, *p *= .01, *η*^2^ = .01 [.001, .03], *χ*^2^(2) = 7.05, *p* = .03 (n.s.)

Pairwise comparisons:

^a^*t*(1, 636) = − 3.10, *p* = .002, *d* = − .25 [− .40, − .09], *z* = − 2.78, *p* = .006

^b^*t*(1, 644) = − .93, *p* = .35, *d* = − .07 [− .23, .08], *z* = − .29, *p* = .78

^c^*t*(1, 636) = 2.04, *p* = .04 (n.s.), *d* = .16 [.01, .32], *z* = 2.40, *p* = .0165

Table 12 reports the ten WoS subject categories with the most HPs and DRs: “Biochemistry & Molecular Biology” (*n *= 68) and “Physics, Multidisciplinary” (*n *= 42) are those categories where most of the papers from both groups belong to. Also, the table reports the results of statistical significance tests for subject category differences between HPs and DRs. There are five statistically significant results. “Biochemistry & Molecular Biology” (HP = 59, DR = 9), “Immunology” (HP = 34, DR = 6), and “Cell Biology” (HP = 22, DR = 4) published more HPs than DRs. In contrast, the subject categories “Surgery” (HP = 3, DR = 37) and “Orthopedics” (HP = 0, DR = 33) are stronger related to DRs than to HPs.

**Table 12:** Ten WoS subject categories with the most HPs and DRs

WoS subject category		HP	DR	Total	*χ*^2^(1)/*p*
Biochemistry & Molecular Biology	Absolut	59	9	68	39.77
	In percent	86.76	13.24	100	**.000**
Physics, multidisciplinary	Absolut	22	20	42	.06
	In percent	52.38	47.62	100	1.00
Immunology	Absolut	34	6	40	20.17
	In percent	85	15	100	**.000**
Surgery	Absolut	3	37	40	31.76
	In percent	7.5	92.5	100	**.000**
Engineering, electrical and Electronic	Absolut	15	20	35	.89
	In percent	42.86	57.14	100	1.00
Orthopedics	Absolut	0	33	33	35.68
	In percent	0	100	100	**.00**
Physics, applied	Absolut	19	8	27	4.40
	In percent	70.37	29.63	100	.36
Cell biology	Absolut	22	4	26	12.53
	In percent	84.62	15.38	100	**.004**
Medicine, general and internal	Absolut	14	6	20	3.1
	In percent	70	30	100	.78
Chemistry, physical	Absolut	9	11	20	.26
	In percent	45	55	100	1.00

The table shows absolute and relative numbers as well as Pearson *χ*^2^ values with Bonferroni-adjusted *p* values (statistically significant results are printed in bold)

## Discussion and conclusions

The existence of DRs has attracted a lot of attention in scientometrics and beyond. The people are fascinated by the fact that researchers publish results which are in advance of one’s time. Studies on DRs dealt either with specific cases of DRs (e.g., Marx 2014) or with methods of detecting DRs (e.g., Ke et al. 2015). Also, citation profiles showing other typical distributions than HPs have been proposed. For example, Ye and Bornmann (2018) define the citation angle distinguishing between HPs and DRs. HPs are highly-cited initially, but the impact decreases quickly. Based on a comprehensive dataset of papers published between 1980 and 1990, we searched for HPs and DRs for further analyses in this study. In contrast to many other studies on DRs, we calculated DNIC values and used these scores for the search of HPs and DRs instead of raw citation counts. In this study, we were interested in identifying systematic differences between HPs and DRs.

The investigation of several variables brought about some interesting results. Since this is the first study investigating differences between HPs and DRs, the results cannot be compared with those of other studies. The investigation of the journals which have published HPs and DRs revealed that some journals (e.g. *Physical Review Letters* and PNAS) were able to publish significantly more HPs than other journals. This pattern did not appear in DRs in this study. Here, the distribution of papers across journals is similar to that in a random sample.

However, this result does not agree to the results of van Raan (2015). He found specific patterns also for DRs. He identified institutions (e.g. MIT) that have more DRs than can be expected based on their relative contribution to the field (in his case: physics). The same was found for journals, particularly *Physical Review B* and *Nuclear Physics B*. Based on the results, van Raan (2015) stated that “a new and interesting question arises whether this type of observations could say something about institutions which are more prone than other institutions to accepting (and publishing) out-of-the-box work”.

In terms of the MNCS (based on single journals or fields), HPs and DRs received impact scores which are significantly above average. However, the citation impact of the DRs is significantly higher than that of the HPs. Many HPs and DRs have been published by authors from the USA; however, in contrast to other countries, authors from the USA have published statistically significantly more HPs than DRs. For other countries, the differences between HPs and DRs are statistically not significant. The WoS subject categories in which the most HPs and DRs have been published are “Biochemistry & Molecular Biology” and “Physics, Multidisciplinary.” Whereas “Biochemistry & Molecular Biology,” “Immunology,” and “Cell Biology” have published significantly more HPs than DRs, the opposite result arrived for “Surgery” and “Orthopedics.” The investigation of HPs and DRs with regard to FICs (e.g., the number of authors) show that HPs have significantly more authors and more (linked) references than DRs/RANs.

The results of this study indicate that especially HPs are differently with respect to certain properties from RANs (e.g. the number of authors), but not necessarily DRs. Our results suggest therefore that the emergence of DRs is an unpredictable process which cannot be fixed by certain properties of the papers. With HPs, this prediction might be possible to a certain extent (Yu et al. 2014). However, this study was a first initial step of analyzing HPs and DRs in comparison. It would be interesting, if future studies address the topic of differences between both groups by using data from other bibliometric databases (especially subject specific databases, as the chemistry-related CA database or the economics RePEc database). These studies could investigate similar variables as those in this study in order to test whether the results of this study can be confirmed. The inclusion of additional variables could reveal further insights in both phenomena: HPs and DRs. Of special interest are variables which cannot be gathered in WoS. So, it could be tested whether the publication of HPs and DRs are related to certain characteristics of authors (e.g. their gender or nationality) or their institutions. Are there certain groups of authors which have published more DRs in the past than can be expected?

In this study, we used field-normalized scores to identify HPs and DRs. Many papers in the WoS database do not only belong to one but so several fields. Thus, it would be interesting to identify those papers in future studies, which are “normal” in one field, but DRs or HPs, respectively, in another.

## Footnotes

1 see http://www.oecd.org/science/inno/38235147.pdf.
